# The efficiency of ^18^F-FDG PET-CT for predicting the major pathologic response to the neoadjuvant PD-1 blockade in resectable non-small cell lung cancer

**DOI:** 10.1007/s00259-020-04711-3

**Published:** 2020-02-11

**Authors:** Xiuli Tao, Ning Li, Ning Wu, Jie He, Jianming Ying, Shugeng Gao, Shuhang Wang, Jie Wang, Zhijie Wang, Yun Ling, Wei Tang, Zewei Zhang

**Affiliations:** 1 Department of PET-CT Center, National Cancer Center/National Clinical Research Center for Cancer/Cancer Hospital, Chinese Academy of Medical Sciences and Peking Union Medical College, Beijing, China; 2 Department of Thoracic Surgery, National Cancer Center/National Clinical Research Center for Cancer/Cancer Hospital, Chinese Academy of Medical Sciences and Peking Union Medical College, Beijing, China; 3 Department of Diagnostic Radiology, National Cancer Center/National Clinical Research Center for Cancer/Cancer Hospital, Chinese Academy of Medical Sciences and Peking Union Medical College, Beijing, China; 4 Department of Pathology, National Cancer Center/National Clinical Research Center for Cancer/Cancer Hospital, Chinese Academy of Medical Sciences and Peking Union Medical College, Beijing, China; 5 Department of Oncology, National Cancer Center/National Clinical Research Center for Cancer/Cancer Hospital, Chinese Academy of Medical Sciences and Peking Union Medical College, Beijing, China

**Keywords:** Non-small cell lung cancer, Checkpoint inhibitors, Neoadjuvant therapy, ^18^F-FDG PET-CT, Pathologic response

## Abstract

**Purpose:**

Investigate whether ^18^F-FDG PET-CT has the potential to predict the major pathologic response (MPR) to neoadjuvant sintilimab in resectable NSCLC patients, and the potential of sifting patients who probably benefit from immunotherapy.

**Methods:**

Treatment-naive patients with resectable NSCLC (stage IA–IIIB) received two cycles of sintilimab (200 mg, intravenously, day 1 and 22). Surgery was performed between day 29 and 43. PET-CT was obtained at baseline and prior to surgery. The following lean body mass–corrected metabolic parameters were calculated by PET VCAR: SUL_max_, SUL_peak_, MTV, TLG, ΔSUL_max_%, ΔSUL_peak_%, ΔMTV%, ΔTLG%. PET responses were classified using PERCIST. The above metabolic information on FDG-PET was correlated with the surgical pathology. (Registration Number: ChiCTR-OIC-17013726).

**Results:**

Thirty-six patients received 2 doses of sintilimab, all of whom underwent PET-CT twice and had radical resection (35) or biopsy (1). MPR occurred in 13 of 36 resected tumors (36.1%, 13/36). The degree of pathological regression was positively correlated with SUL_max_ (*p* = 0.036) of scan-1, and was negatively correlated with all metabolic parameters of scan-2, and the percentage changes of the metabolic parameters after neoadjuvant therapy (*p* < 0.05). According to PERCIST, 13 patients (36.1%, 13/36) showed partial metabolic response (PMR), 21 (58.3%, 21/36) had stable metabolic disease, and 2 (5.6%, 2/36) had progressive metabolic disease (PMD). There was a significant correlation between the pathological response and the PET responses which were classified using PERCIST. All (100.0%) the PMR (ΔSUL_peak_% < − 30.0%) tumors showed MPR.

**Conclusions:**

^18^F-FDG PET-CT can predict MPR to neoadjuvant sintilimab in resectable non-small cell lung cancer.

**Electronic supplementary material:**

The online version of this article (10.1007/s00259-020-04711-3) contains supplementary material, which is available to authorized users.

## Introduction

Worldwide, non-small cell lung cancer (NSCLC) is the most common cause of cancer death [1, 2], and more than 70% of patients are found as locally advanced or metastatic disease at the time of diagnosis. Immunotherapy has become a new therapeutic approach in NSCLC with the potential for prolonged benefits [3]. Since 2015, three immune checkpoint inhibitors (nivolumab, pembrolizumab, and atezolizumab) have been approved by the US Food and Drug Administration (FDA) for the treatment of NSCLC [4].

Selecting patients who will benefit before or at the early stage of immunotherapy is a major issue in clinical application [5]. The expression level of programmed death ligand 1 (PD-L1) in NSCLC is chiefly used to screen patients for immunotherapy in clinical trials. However, the patients who cannot obtain sufficient specimens for subjective or objective reasons usually cannot complete the pathological test, even more impossible to gain specimens repeatedly to evaluate curative efficacy. Furthermore, the tumor response patterns of immunotherapy may differ compared with conventional chemotherapeutic agents or targeted therapies, and the accuracy of response assessment is radiologically challenging [6–8].

^18^F-FDG PET-CT is the most useful tool for evaluating changes of lesion on molecular level. The mechanism of FDG uptake within tumor cells is concerned with the presence of glucose metabolism, hypoxia, and angiogenesis [9–11]. The level of PD-L1 expression has been associated with that of glucose transporter 1 (Glut1) and hypoxia-inducible factor 1α (HIF-1α) in patients with NSCLC [12, 13]. Lopci et al. [14] found a direct association between SUV_max_ and SUV_mean_ with the expression of PD-1 (rho = 0.33; *p* = 0.017 and rho = 0.36; *p* = 0.009, respectively) in patients with NSCLC. In a recent study, FDG PET was considered to provide more useful information on assessing response of advanced NSCLC to immunotherapy than that of computed tomography (CT) [15]. On the basis of these findings, ^18^F-FDG PET-CT in predicting immunotherapy response to NSCLC seems to be a valuable and important research area in clinical application. Sintilimab is a recombinant humanized anti-PD-1 monoclonal antibody injection that blocks interactions between PD-1 and its ligands and has been tested regarding the safety and activity in patients with advanced stage solid tumor and was approved for lymphoma by Chinese Center for Drug Evaluation in China in 2018 [16]. Phase I/II development of sintilimab for use in solid tumors is underway in the USA, with the US FDA accepting an Investigational New Drug Application for sintilimab in January 2018 [16]. The current study aims to evaluate the relationship between tumor metabolic parameters of ^18^F-FDG PET-CT and the surgical pathology of the neoadjuvant sintilimab in resectable NSCLC patients, and to investigate if PET-CT has the potential to predict the major pathologic response (MPR), which predict improved long-term patient outcome [17, 18].

## Materials and methods

### Patients and methods

The study was a prospective, single-center, single-arm, phase Ib study (Registration Number: ChiCTR-OIC-17013726). The Ethics Committee and Institutional Review Board of National Cancer Center/ Cancer Hospital, Chinese Academy of Medical Sciences and Peking Union Medical College approved this prospective study and written informed consent was obtained from patients before PET/CT examinations. Eligible patients were 18–75 years of age and had histologically or cytologically confirmed NSCLC (stage IA–IIIB, AJCC 8th) [19] that was surgically resectable. All patients were treatment-naive and had a primary tumor with diameter ≥ 2 cm, an Eastern Cooperative Oncology Group Performance Status of 0, and adequate organ function. Exclusion criteria were epidermal growth factor receptor (EGFR)–sensitive mutation; previous anti-tumor therapy; systemic immunosuppressive therapy within 4 weeks prior to study treatment; known or suspected active autoimmune diseases; previous allogeneic organ transplantation or hematopoietic stem cell transplantation; hypersensitive to any monoclonal antibodies; history of interstitial lung disease; active and uncontrolled infection; grade III–IV congestive heart failure; uncontrolled hypertension; uncontrolled hypercalcemia; artery thrombosis, embolism, or ischemia within 6 months prior to study treatment; coagulation disorders requiring warfarin treatment; other known malignant tumor. The complete eligibility criteria are shown in the Supplementary materials (Inclusion criteria and Exclusion criteria).

Contrast-enhanced CT or magnetic resonance imaging (MRI) was performed to exclude brain metastases at baseline. PET-CT was performed at baseline (scan-1) and within 1 week prior to surgery (scan-2). PET responses were classified using PERCIST criteria [20].

The patients who were eligible to this clinical trial received two cycles of sintilimab (200 mg, intravenously, day 1 and 22). Complete tumor resection or biopsy (confirming tumor progression) would be performed approximately 29–43 days after the first dose. Primary tumors were assessed for the percentage of residual viable tumor in the resected lung tissue, and MPR defined as tumors with no more than 10% viable tumor cells [18].

### FDG PET-CT acquisition

PET-CT was performed from head to thigh using an integrated PET-CT (Discovery 690, GE Healthcare). All patients were instructed to fast for at least 6 h before scan. Blood glucose levels were required to be < 145 mg/dl. Patients were injected intravenously with a mean dose of 3.70–4.44 MBq/kg of ^18^F-FDG. The differences in injected doses of ^18^F-FDG was less than 20%, and the differences in uptake time was less than 15 min between the scan-1 and scan-2. Whole-body 3-dimensional PET-CT scan was acquired 60  min after ^18^F-FDG injection. The PET images were obtained with 2 min per frame in the three-dimensional mode from head to the upper femur (generally 7–8 beds location). Images were reconstructed using the VPFX-S algorithm (2 iterations, 24 subsets, 4 mm Gaussian post-filter). Spiral CT was performed with a tube voltage of 120 kV, tube current of 150 mA, 1.375 of pitch, 3.75 mm of slice thickness, and 0.8 s of rotation speed.

### Image analysis

All images were observed and analyzed using PETVCAR, which is an automated segmentation software system by using an Advantage Workstation (version 4.6; GE Healthcare). A volume-of-interest (VOI) around the whole tumor was auto-contoured and segmented using a boundary box, which was placed by two experienced radiologists of PET-CT center who adjusted to ensure this 3-dimensional cube contained all the FDG PET positive area and excluded the negative normal tissue in either of the axial, sagittal, and coronal planes by consensus. Both of the two radiologists were unaware of the patient’s clinical history and data. The following lean body mass–corrected metabolic parameters were calculated by PETVCAR: SUL_max_, SUL_peak_, metabolic tumor volume (MTV), total lesion glycolysis (TLG). According to PERCIST [20], the SUL_peak_ of each evaluable lesion was at least 1.5 × (SUL_mean_ (live) + 2SD), and the treatment response was evaluated by percentage changes of the highest intensity (ΔSUL_peak_%). The post-treatment percentage changes of metabolic parameters calculated by PETVCAR were recorded. The formula was as follows take ΔSUL_max_% for example, ΔSUL_max_% = (SUL_max_ of scan-1 − SUL_max_ of scan-2)/SUL_max_ of scan-1 × 100%. Response to the neoadjuvant therapy was classified as (1) complete metabolic response (CMR), defined as a complete resolution of ^18^F-FDG uptake within the measurable target lesions and other lesions (less than mean liver activity and at the level of surrounding background blood-pool activity) without the advent of new suggestive ^18^F-FDG avid lesions; (2) partial metabolic response (PMR), defined as a reduction of 30% or more in the target tumor SUL_peak_ (and an absolute drop of at least 0.8 SUL); (3) progressive metabolic disease (PMD), defined as 30% or more increase in SUL_peak_ and 0.8 unit increase in SUL_peak_ or the advent of new ^18^F-FDG avid lesions typical of cancer; (4) stable metabolic disease (SMD), defined as disease other than CMR, PMR, or PMD [20].

### Pathological assessments

Primary lung tumor and lymph-node surgical specimens were staged according to the criteria of the American Joint Committee on Cancer (AJCC 8th) for evaluating tumor size, affected lymph nodes, and metastases [19]. Primary tumors were assessed for the percentage of residual viable tumor that was identified on routine hematoxylin and eosin staining, and tumors with no more than 10% viable tumor cells were considered to have MPR.

### Statistical analysis

Patients with MPR were further classified as responders; the patients without MPR were classified as non-responders. Statistical Product and Service Solutions (SPSS, version 17.0) was used for data analysis. All data were verified for normal distribution with Kolmogorov-Smirnov test and for homogeneity of variance with Levene test. Data for SUL_max_, SUL_peak_, ΔSUL_peak_%, and ΔSUL_peak_% were approximately normally distributed, while data for MTV, TLG, ΔMTV%, and ΔTLG% were not normally distributed. These data are presented here in terms of mean ± standard deviation (SD), median, and range. The independent sample *t* test was used to compare SUL_max_ with SUL_peak_ between responders and non-responders, while the Mann-Whitney *U* test (MW) was used to compare MTV and TLG between the two groups. The relationship between metabolic parameters and the percentage of residual viable tumor in the resected primary tumor after neoadjuvant therapy was evaluated by Pearson’s correlation analysis or Spearmen’s correlation analysis, depending on the data whether or not conform to normal distribution. The value of parameters on predicting responders were calculated by receiver operating characteristic curve (ROC). Statistical significance was set at *p* < 0.05.

## Results

### Characteristics of patients

From March 6, 2018 to March 8, 2019, a total of 40 patients with NSCLC, all of whom received 2 doses of sintilimab as neoadjuvant treatment, were enrolled in the clinical trail and 36 were finally enrolled in this study. Four patients were excluded. Two of them were excluded for the baseline PET-CT which was underwent in other hospital, while two patients, classified as SMD according to PERCIST, were excluded for the pathological regression of the primary tumor could not be assessed by the exploratory surgery. Among 36 enrolled patients (29 men, 7 women; median age 61 years, range 48–70 years), most patients (80.6%, 29/36) were squamous cell carcinoma, and the mean tumor size was (4.7 ± 1.5 cm; range 2.3 cm–7.4 cm). The characteristics of patients were summarized in Table 1.

### Pathological and metabolic findings after neoadjuvant sintilimab

The median degree of pathological regression in the primary tumor was 42.5% (0–100%). Thirteen patients (36.1%, 13/36) had MPR, which were all with squamous cell NSCLC. Five patients (13.9%, 5/36) had complete pathological response (pCR) of primary tumor, and two patients (5.6%, 2/36) obtained pCR in both primary tumor and lymph nodes. Twenty-two patients (61.1%, 22/36) did not have MPR, but had varying degrees of pathological regression. All pathological and metabolic findings after neoadjuvant sintilimab of 36 enrolled patients are shown in the Supplementary materials (Table S). One patient (2.8%, 1/36) was confirmed as tumor progression by the biopsy of a new metastasis on pleural. There was no association between baseline characteristics and MPR in terms of age, gender, histology, smoking history, clinical stage.

SUL_max_ of scan-1 was positively correlated (*p* = 0.036) with the degree of pathological regression of primary tumor. All metabolic parameters of scan-2 and the percentage changes of metabolic parameters after neoadjuvant therapy were negatively correlated (*p* < 0.05) with the tumor regression (Table 2). The characteristics of metabolic parameters between responders and non-responders were summarized in Table 3. ROC indicated that ΔSUL_max_% and ΔSUL_peak_% had the best differentiation ability (Table 4). By setting threshold of ΔSUL_max_% and ΔSUL_peak_% both to − 30%, the specificity, sensitivity, and accuracy were 100%, 100%, and 100%, with area under curve (AUC) of 1 (*p* = 0.000).

### Correlation between metabolic response and pathological response

**Fig. 1:**
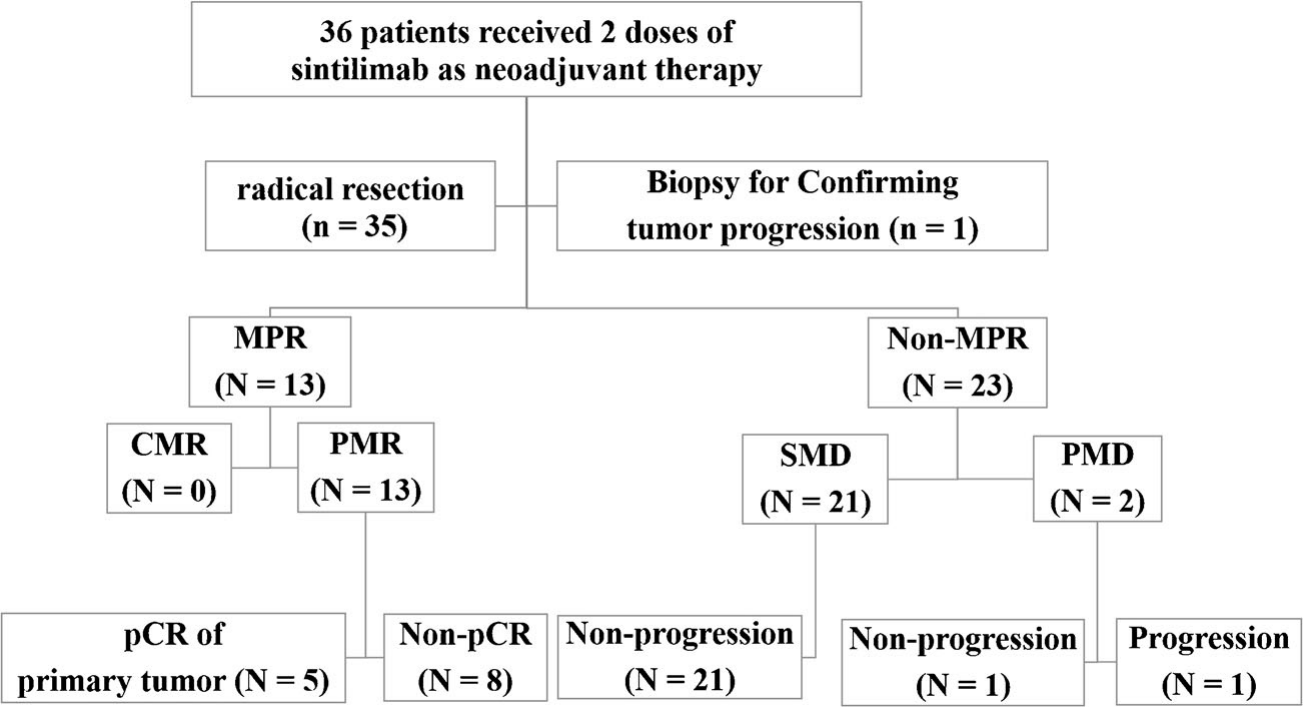
Characteristics of metabolic response according to pathological response (CMR, complete metabolic response; PMR, partial metabolic response; SMD, stable metabolic disease; PMD, progressive metabolic disease. Progression was confirmed by the biopsy of a new metastasis on pleural; pCR, a complete pathological response of primary tumor; Three patients with pCR of primary tumor had residual tumor in mediastinal lymph nodes)

**Fig. 2:**
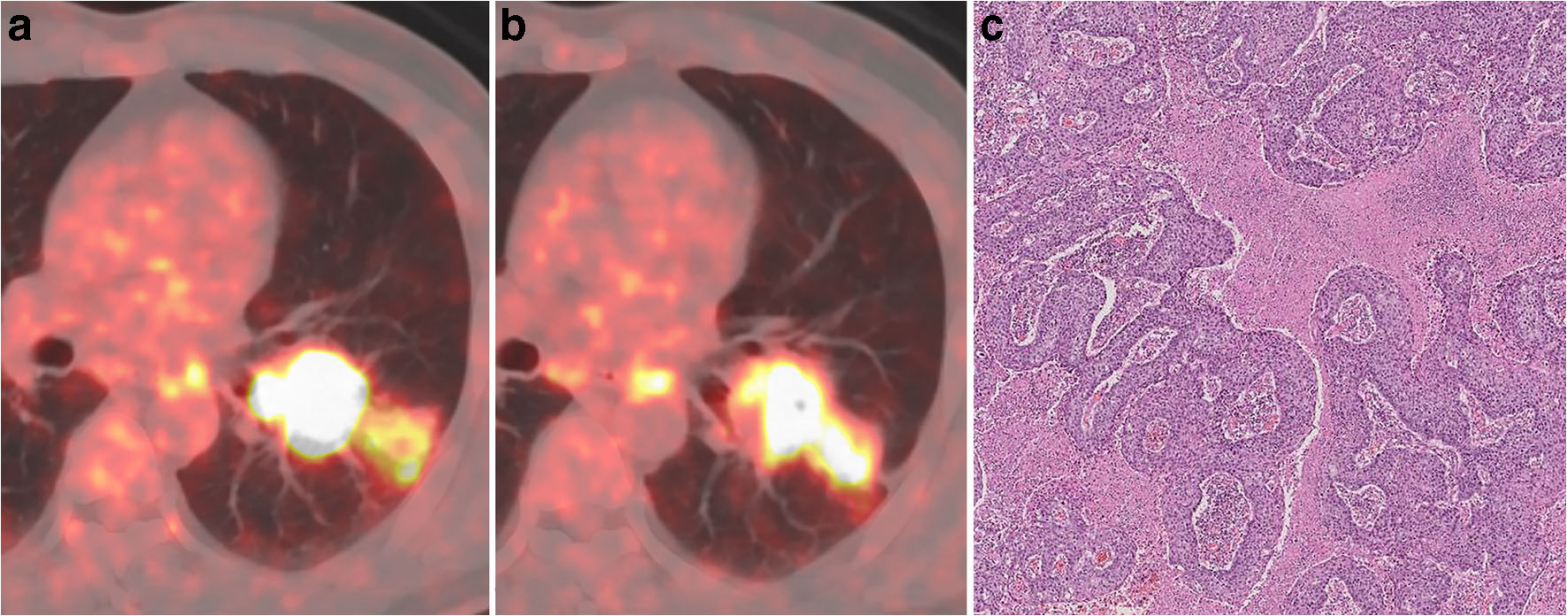
A 49-year-old man with squamous cell lung cancer, who had no marked morphologic changes on CT where evaluated as PMR according to PERCIST after two doses sintilimab treatment, was shown. **a** Axial fusion image of scan-1, SUL_peak_ = 19.3. **b** Axial fusion image of scan-2, SUL_peak_ = 11.8; ΔSUL_peak_% = − 38.7%. **c** Resection specimen showed this patient had MPR (less than 10% residual viable tumor)

**Fig. 3:**
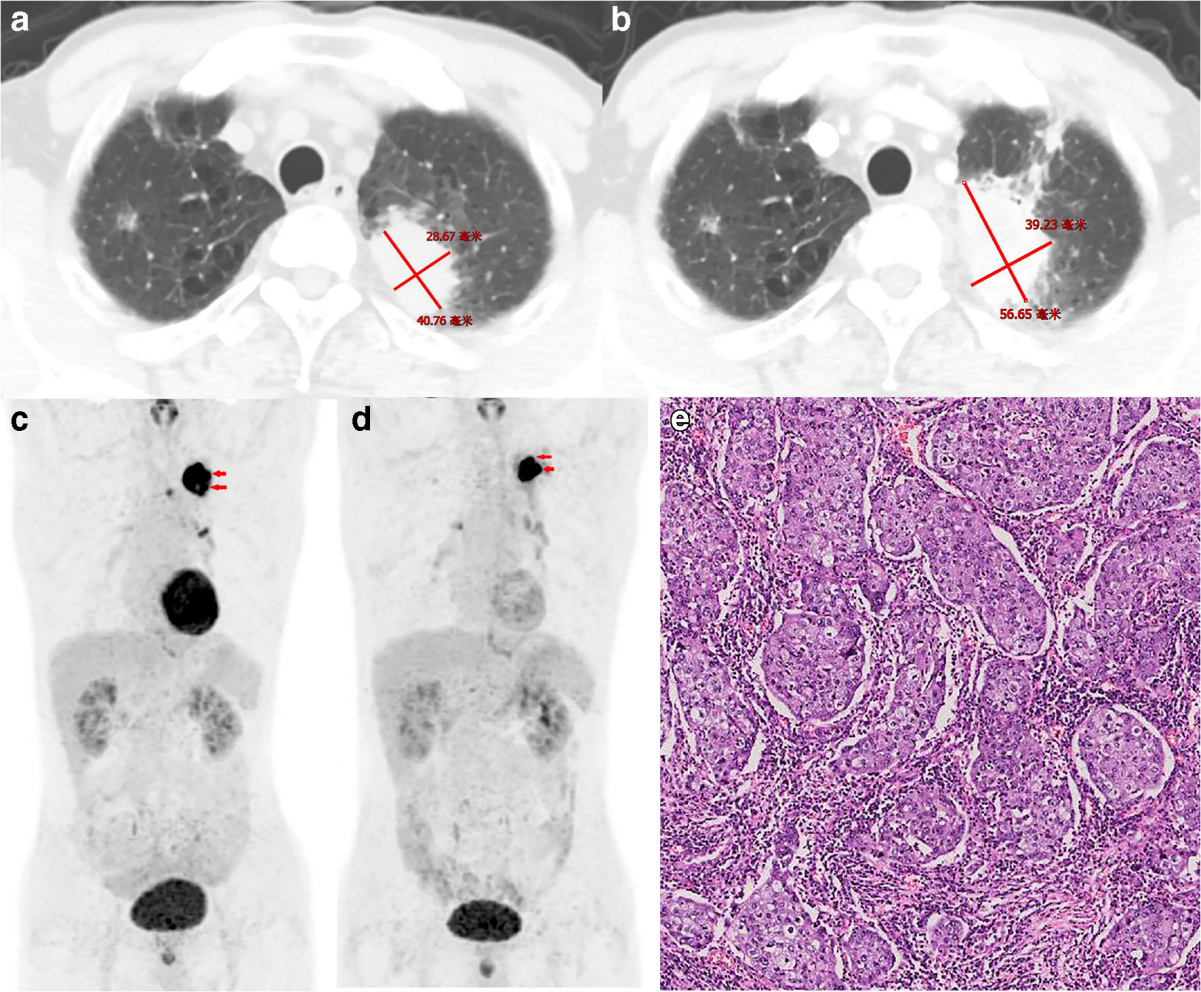
A 65-year-old man with lung adenocarcinoma, who evaluated as PMD according to PERCIST after two doses sintilimab treatment, was shown. **a** Contrast-enhanced axial CT of scan-1. **b** Contrast-enhanced axial CT of scan-2 showed this patient had a remarkable enlargement in the size of tumor than that of scan-1. **c** MIP (maximum intensity projection) image of scan-1, SUL_peak_ = 11.4; MTV = 24.3; TLG = 194.8. **d** MIP image scan-2: Despite SUL_peak_ of this patient on scan-2 (SUL_peak_ = 15.4) had a conductivity increase (ΔSUL_peak_% = 32.1%), either ΔMTV% or ΔTLG% of the primary tumor (red arrow) were conductivity decreased (MTV = 9.7, ΔMTV% = −60.1%; TLG = 97.4, ΔTLG% = − 50%). **e** Resection specimen showed this patient had 60% of pathological regression and were observed large numbers of macrophages and infiltrating lymphocytes

**Fig. 4:**
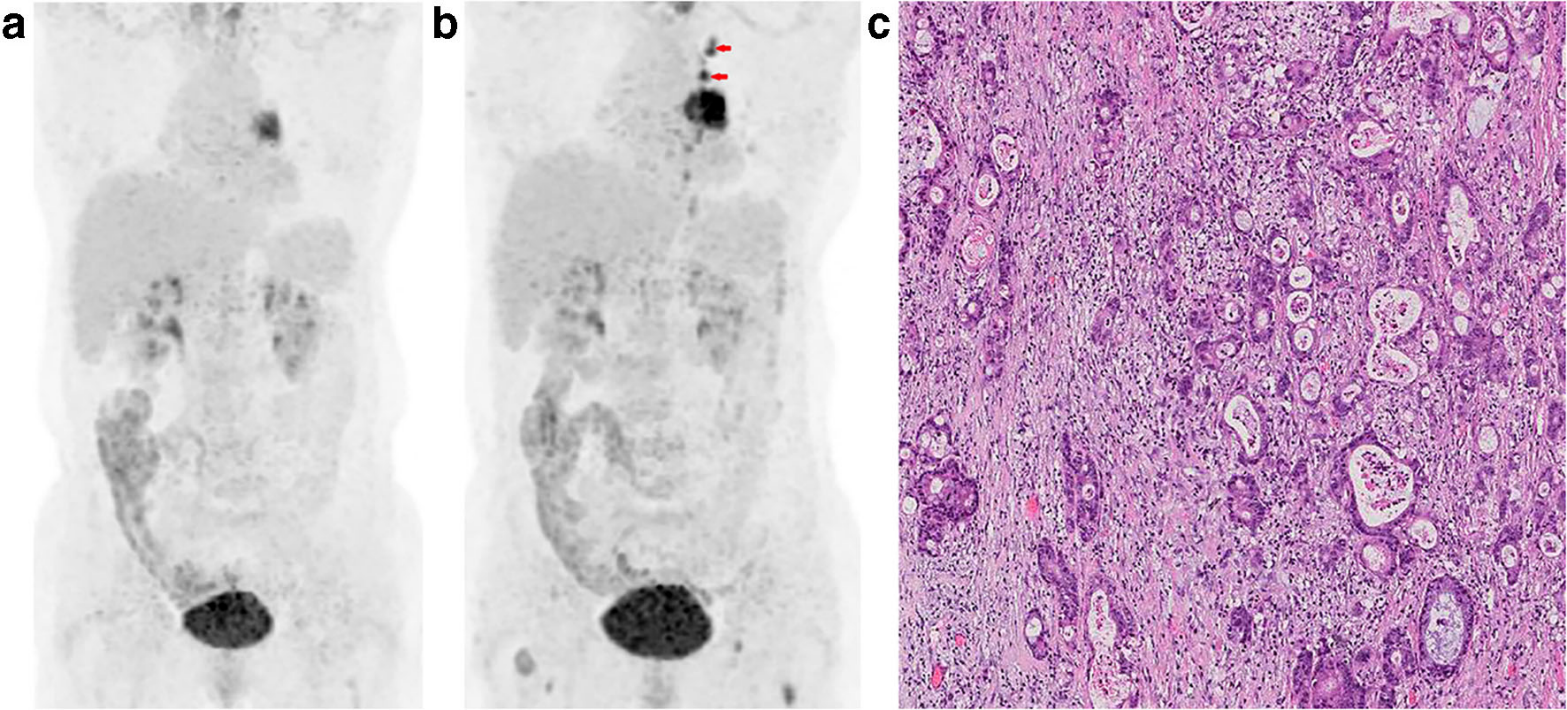
A 64-year-old woman with lung adenocarcinoma, who had new metastases on pleural (red arrow) and evaluated as PMD according to PERCIST after two doses sintilimab treatment, was shown. **a** MIP (maximum intensity projection) image of scan-1, SUL_peak_ = 4.7. **b** MIP image of scan-2: All metabolic parameters of this patient on scan-2 had a conductivity increase (ΔSUL_peak_% = 59.4%, ΔSUL_max_% = 77.3%, ΔMTV% = 100%, ΔTLG% = 205.1%). **c** Pleural biopsy confirmed this patient had metastases on pleural

According to PERCIST, the metabolic response to sintilimab of all patients were classified as CMR (0%, 0/36), PMR (36.1%, 13/36), SMD (58.3%, 21/36), or PMD (5.6%, 2/36). All patients with PMR (100%, 13/13) had MPR, including 5 pCR (38.5%, 5/13) of primary tumor (Fig. 1). Six patients (46.2%, 6/13) with PMR (4 of them had MPR, and 2 of them had pCR of primary tumor) were observed that the size of tumor had no remarkable changes on preoperative PET-CT (Fig. 2). Both of the two PMD patients had a remarkable enlargement in the size of tumor on preoperative PET-CT. One PMD patient was observed to have large numbers of macrophages and infiltrating lymphocytes, and had 60% of pathological regression of primary tumor (Fig. 3). The other PMD patient was confirmed as progression by the biopsy of a new metastasis on pleural and had a conductivity increase in all metabolic parameters of scan-2 (Fig. 4).

## Discussion

Immunotherapy is one of the most exciting fields in NSCLC with the potential for prolonged benefits [3]. Evaluation of this novel therapy is a major challenge, since immunotherapy radically differs from other strategies in relying on the reactivation of the immune system to recognize and kill cancer cells [21]. The use of immunomodulatory monoclonal antibodies that directly enhance the function of components of the antitumor immune response, such as T cells, or block immunological checkpoints that would otherwise restrain effective antitumor immunity has recently been actively investigated in oncology [21, 22]. Forde et al. [23] reported that a major pathological response occurred in 45% of tumors after neoadjuvant administration of two doses of nivolumab in patients with early stage lung cancer, and two patients whose tumors had increased in size on presurgical CT scans (although the increase was less than RECIST-defined progression) were found to have minimal or no residual tumor in the surgical specimen. These findings represent pathological evidence supporting the possibility that some patients may derive clinical benefit from immunotherapy without initial radiographic tumor shrinkage. Conventional imaging criteria, either RECIST1.1 or iRECIST, has the above limitations for depending on morphologic changes [6–8, 24]. PET-CT was considered to overcome such limitations and more suitable for assessment of therapeutic effect, because it can reflect on tumor metabolic level before morphological changes. In 2009, Wahl et al. [20] proposed the PET Response Criteria in Solid Tumors. The major concepts of PERCIST were the use of SUL for the tumor response assessment, and the identification of a measurable target lesion SUL is at least 1.5-fold greater than liver SUL_mean_ + 2 SDs (in 3 cm spherical ROI in normal right lobe of liver) [20]. PERCIST proposed to use the percentage change in SUL_peak_ (or sum of lesion SULs) between the pre- and post-treatment scans for assessing therapy response. The mechanism of FDG uptake within tumor cells is concerned with the presence of glucose metabolism, hypoxia, and angiogenesis [9–11]. The level of PD-L1 expression is associated with Glut1 and HIF-1α in patients with NSCLC [12, 13]. Therefore, some studies attempt to search whether baseline ^18^F-FDG PET-CT can provide useful information on the expression of checkpoint inhibitors in patients with NSCLC, in order to distinguish patients from the potential for prolonged benefits. Indeed, there is a statistically significant association between tumor metabolic parameters on ^18^F-FDG PET-CT and PD1/PD-L1 expression in resected tumor specimens [12–14, 25]. Grizzi et al. [26] found that almost all patients (*n* = 27) with SUV_max_ ≤ 17.1 or SUV_mean_ ≤ 8.3 on baseline PET had fast progression after 8 weeks immunotherapy.

Our study has revealed the clinical significance of ^18^F-FDG PET-CT as a promising biomarker for predicting early phase clinical outcomes of PD-1 blockade therapy in NSCLC patients. Specially, PMR (100%, 13/13) according to PERCIST showed excellent prediction capabilities to distinguish patients with MPR. Despite metabolic parameters of baseline ^18^F-FDG PET-CT, including SUL_max_, SUL_peak_, MTV, TLG, cannot distinguish patients with MPR (*p* > 0.05), which may be due to small-sized sample, SUL_max_ and SUL_peak_ of baseline were positively correlated to the degree of pathological regression (SUL_max_, *p* = 0.036; SUL_peak_, *p* = 0.058). The result may indirectly support the hypothesis that metabolic characteristics of tumor on baseline may be part of a larger panel of predictive factors of response to immunotherapy of NSCLC [22, 27].

Although little is known about PERCIST criteria with respect to response to immunotherapy of NSCLC, there are a few studies or case reports describing its role in evaluating response to immune checkpoint inhibitors [15, 25, 28–30]. In a recent study, 24 patients treated with PD-1 blockade (nivolumab) were investigated at baseline and 1 month after the start of treatment [15]. Response was determined using both morphological (RECIST 1.1) and PERCIST criteria. The value of PET in predicting PR (partial response) and PD (progressive disease) was significantly higher than that of CT. The multivariate analysis confirmed FDG uptake after administration of nivolumab was an independent prognostic factor in predicting progression free survival (PFS) (HR 3.624, *p* < 0.001) and overall survival (OS) (HR 2.461, *p* = 0.012) [15]. Another study assessing response of NSCLC to immunotherapy, 103 patients treated with anti-PD-L1 agent (atezolizumab) were evaluated the potential of FDG PET-CT for assessing response [30]. Patients with metabolic response on 6-week scans had a higher ORR (objective response rate) than metabolic non-responders (73.9% (in 17 of 23 patients) vs. 6.3% (in 5 of 80 patients)) [30]. The reports above have noted that PET-CT is a useful tool for immune monitoring, nevertheless, the optimal time for evaluating the appropriate efficacy remains uncertain. The results of our study suggest that 4 weeks after the first dose of sintilimab maybe an opportune moment, and the percentage changes of the metabolic parameters (SUL_peak_%, ΔSUL_max_%) could correctly predict MPR. The specificity, sensitivity, and accuracy were 100%, 100%, and 100% (*p* = 0.000). The metabolic parameters of scan-2 also showed good prediction capabilities to distinguish patients with MPR. All metabolic parameters of scan-2, including SUL_max_, SUL_peak_, MTV, TLG, were negatively correlated with the degree of pathological regression. SUL_peak_ of scan-2 has the best differentiation ability. By setting threshold of SUL_peak_ to 6.7, the specificity, sensitivity, and accuracy were 92.3%, 81.8%, and 86.1%, with AUC of 0.90 (*p* = 0.000). This preliminary result of our study demonstrated the clinical significance of the follow-up scan at 4 weeks after the first dose of sintilimab treatment and may provide useful information for selecting patients who had benefit at the early stage of immunotherapy.

In addition, we observed an interesting phenomenon in this study. One patient (Fig. 3) in this clinical trial was evaluated as PMD before surgery. He had a remarkable enlargement in the size of tumor (4.1 cm vs. 5.7 cm) on preoperative PET-CT. Despite ΔSUL_peak_% (32.1%) had a significant increase, both ΔMTV% (− 60.1%) and ΔTLG% (− 50%) of the primary tumor decreased markedly. The postoperative pathology showed the primary tumor had 60% of pathological regression and large numbers of macrophages and infiltrating lymphocytes. This interesting phenomenon may help to explain the pathological basis of pseudoprogression [22, 24]. Besides, the deviation of metabolic parameters (e.g., increased ΔSUL_peak_% vs. reduced ΔMTV% and ΔTLG%) may help to differentiate pseudoprogression from PMD. However, there was only one patient with pathologically confirmed “pseudoprogression” according to PERCIST criteria. Further studies are necessarily needed to explore the efficiency of the combined application of multiple metabolic parameters for distinguishing “pseudoprogression” from PMD.

There are several limitations in this study. Firstly, our study is a preliminary study and includes a small sample size. There was only one patient with histopathologically confirmed PD. Thus, we did not analyze the potential of metabolic parameters to predict patients who cannot benefit from immunotherapy before surgery. Further studies including larger numbers of patients are necessary to validate these results. Secondly, this study mainly focuses on the metabolic response of ^18^F-FDG PET-CT for predicting the major pathologic response to the neoadjuvant PD-1 blockade. We did not analyze the relationship between biomarkers such as the tumor mutational burden and PD-L1 expression and metabolic parameters, for they are not available yet until we submit the manuscript. We also did not analyze the immune-related side effects in this study. Thirdly, previous studies indicated that EGFR mutations were associated with low response rates to PD-1 blockade treatment among patients with NSCLC; in some cases, inhibition of checkpoint blockade even increased the rate of tumor growth considerably [31–33]. Therefore, we excluded the patients with the presence of EGFR-sensitive gene mutation in tumor tissue. However, EGFR mutation rate are very high (51.8%) in Chinese lung adenocarcinoma population [34], and quite a few (56%) adenocarcinoma were ground glass opacity (GGO) which were not suitable for the study [35]. Therefore, most of the patients with adenocarcinoma were excluded, and the large majority of the patients had squamous cell carcinoma subtype in this trial, which may bias the results in this study. Fourthly, we did not evaluate clinical end points such as OS rate or PFS, as our study focused on MPR, which strongly associates with improved survival of neoadjuvant therapy [17, 18]. Long-term follow-up is necessary to confirm the prognostic significance of OS using ^18^F-FDG PET-CT.

## Conclusions

Metabolic responses by ^18^F-FDG uptake which were classified using PERCIST are significantly associated with therapeutic response at 4 weeks after two doses sintilimab treatment, and can predict MPR to the neoadjuvant therapy in resectable NSCLC. The metabolic parameter of PET-CT appears to be a promising biomarker for screening patients who probably benefit from immunotherapy.

### Electronic supplementary material

ESM 1 (DOCX 42 kb)

## Appendix

**Table 1:** Characteristics of the patients according to pathological response

Characteristic	All patients (*N* = 36)	Patients with MPR (*N* = 13)	Patients without MPR (*N* = 23)
Age (year)			
Median age, year (range)	61 (48–70)	61 (49–70)	61 (48–70)
Sex, no. (%)			
Female	7 (19.4)	1 (7.7)	6 (26.1)
Male	29 (80.6)	12 (92.3)	17 (73.9)
Histologic diagnosis, no. (%)			
Adenocarcinoma	6 (16.7)	0 (0.0)	6 (26.1)
Squamous cell carcinoma	29 (80.6)	13 (100.0)	16 (69.6)
Mixed	1 (2.8)	0 (0)	1 (4.3)
Clinical stage, no. (%)			
Ia	1 (2.8)	0 (0)	1 (4.3)
Ib	6 (16.7)	1 (7.7)	5 (21.8)
IIb	12 (33.3)	4 (30.8)	8 (34.8)
IIIa	9 (25.0)	6 (46.2)	3 (13.0)
IIIb	8 (22.2)	2 (15.3)	6 (26.1)
Smoking status, no. (%)			
Never	8 (22.2)	1 (7.7)	7 (30.4)
Former or current	28 (77.8)	12 (92.3)	16 (69.6)

*MPR* major pathological response, defined as the identification of 10% or less of residual viable tumor cells in the resected primary tumor

The clinical stage before neoadjuvant therapy was evaluated according to the criteria of the American Joint Committee on Cancer, eighth edition

The eight patients of IIIb were T3N2aM0 (6 patients) or T4N2aM0 (2 patient) according to AJCC 8th

**Table 2:** Correlation between metabolic parameters and the degree of pathological regression in the resected primary tumor after neoadjuvant therapy

Metabolic parameters	Degree of pathological regression	
	*r* value	*p* value
Scan-1		
SUL_max_	0.351	0.036
SUL_peak_	0.319	0.058
MTV	− 0.083	0.630
TLG	− 0.028	0.879
Scan-2		
SUL_max_	− 0.503	0.002
SUL_peak_	− 0.577	0.000
MTV	− 0.452	0.006
TLG	− 0.578	0.000
The percentage changes(Δ%) between scan-1 and scan-2		
ΔSUL_max_%	− 0.837	0.000
ΔSUL_peak_%	− 0.874	0.000
ΔMTV%	− 0.696	0.000
ΔTLG%	− 0.886	0.000

The relationship between SUL_max_, SUL_peak_, ΔSUL_peak_% and ΔSUL_peak_%, and the percentage of residual viable tumor after neoadjuvant therapy was evaluated by Pearson’s correlation analysis, while MTV, TLG, ΔMTV%, and ΔTLG% was evaluated by Spearmen’s correlation analysis

**Table 3:** Characteristics of metabolic parameters according to pathological response

Metabolic parameters	Responders (*N* = 13)	Non-responders (*N* = 23)	*t*/*z* value	*p* value
	Mean ± SD or median and range	Mean ± SD or median and range		
Scan-1				
SUL_max_	16.9 ± 6.7	12.9 ± 4.5	− 1.942	0.068
SUL_peak_	12.5 ± 5.1	9.6 ± 3.5	− 2.008	0.053
MTV	18.4 (1.7, 108.0)	24.5 (5.0, 222.0)	− 0.949	0.626
TLG	240.1 (7.7, 1069.0)	151.7 (17.2, 1543.8)	− 0.049	0.974
Scan-2				
SUL_max_	6.7 ± 4.3	12.8 ± 4.6	3.952	0.000
SUL_peak_	4.3 ± 2.8	9.8 ± 3.5	4.838	0.000
MTV	10.4 (1.0, 55.4)	24.3 (5.9, 186.0)	− 2.306	0.020
TLG	33.8 (2.0, 156.1)	125.4 (16.3, 1222.8)	− 3.277	0.001
The percentage changes(Δ%) between scan-1 and scan-2				
ΔSUL_max_%	− 61.9 ± 13.7	1.7 ± 23.1	9.049	0.000
ΔSUL_peak_%	− 66.9 ± 13.1	3.5 ± 19.9	11.361	0.000
ΔMTV%	− 50.0 (− 91.0, − 27.0)	− 24.0 (− 91.0, 100.0)	− 4.117	0.000
ΔTLG%	− 85.4 (− 96.9, − 68.1)	− 12.3 (− 69.6, 205.1)	− 4.891	0.000

Data for SUL_max_, SUL_peak_, ΔSUL_peak_%, and ΔSUL_peak_% were approximately normally distributed, and the independent sample *t* test was used to compare metabolic parameters above between responders and non-responders. These data are presented here in terms of mean ± standard deviation (SD), *t* value and *p* value

Data for MTV, TLG, ΔMTV%, and ΔTLG% were not normally distributed, the Mann-Whitney *U* test was used to compare metabolic parameters above between responders and non-responders. These data are presented here in terms of median, range, *z* value and *p* value

**Table 4:** Values of the metabolic parameters on predicting responders

Metabolic parameters	Threshold	AUC	Sensitivity (%)	Specificity (%)	Accuracy (%)
Scan-2					
SUL_max_	7.9	0.84	76.9	90.9	86.1
SUL_peak_	6.7	0.90	92.3	81.8	86.1
MTV	16.4	0.74	69.2	69.6	69.4
TLG	87.1	0.84	76.9	73.9	75.0
The percentage changes(Δ%) between scan-1 and scan-2					
ΔSUL_max_%	− 30.0%	1.00	100.0	100.0	100.0
ΔSUL_peak_%	− 30.0%	1.00	100.0	100.0	100.0
ΔMTV%	− 33.0%	0.92	92.3	82.6	86.1
ΔTLG%	− 60.0%	0.99	100.0	95.7	97.2
