# Supplementary material for: The efficiency of 18F-FDG PET-CT for predicting the major pathologic response to the neoadjuvant PD-1 blockade in resectable non-small cell lung cancer
Source: Eur J Nucl Med Mol Imaging. 2020 Feb 11;47(5):1209–19. doi: 10.1007/s00259-020-04711-3 (PMC7101299; doi:10.1007/s00259-020-04711-3)
Supplement: Supplementary file 1 — (DOCX 42 kb) [file 259_2020_4711_MOESM1_ESM.docx]

**Supplementary Materials:**

Inclusion criteria and Exclusion criteria

**Inclusion criteria:**

1. Cytologically or histologically confirmed NSCLC;

2. Assent to the collection of tumor histological specimens required for this study for relevant studies;

3. Presence of treatment-naïve, surgically resectable NSCLC, with a tumor diameter of > 2 cm;

4. Consent to receive radical operation;

5. No surgical contraindications in the judgment of a thoracic surgeon;

6. Presence of at least one measurable lesion (RECIST v1.1);

7. Male or female, at an age ≥ 18 years and ≤ 75 years;

8. An ECOG score of 0;

9. Adequate functions of vital organs and bone marrow that meet the following requirements:

-Hematology: absolute neutrophil count (ANC) ≥ 1.5 × 10^9^/L, platelets (PLT) ≥ 100 × 10^9^/L, hemoglobin (HGB) ≥ 9 g/dL;

-Liver function: serum total bilirubin (TBIL) ≤ 1.5 × ULN (upper limit of normal), alanine aminotransferase (ALT) and/or aspartate aminotransferase (AST) ≤ 2.5 × ULN, serum albumin (ALB) ≥ 2.8 g/dL;

-Renal function: Serum creatinine (Cr) ≤ 1.5 × ULN, or creatinine clearance ≥ 40 mL/min (calculated using the standard Cockcroft -Gault formula):

Females: CrCl = (140-age) x body weight (kg) x 0.85

72 x serum creatinine (mg/dL)

Males: CrCl = (140-age) x body weight (kg) x 1.00

72 x serum creatinine (mg/dL)

10. Provide written ICF and could follow the visit schedule and relevant procedures specified in the study protocol.

**Exclusion criteria:**

1. Presence of EGFR-sensitive gene mutation in tumor tissue as revealed by aspiration biopsy;

2. A past medical history of any anti-tumor therapy, including radiotherapy, chemotherapy, immunotherapy and traditional Chinese medicine therapy (excluding therapy for malignancies that were radically treated, without recurrence or metastasis for a period of ≥5 years);

3. Use of immunosuppressive agents within 4 weeks prior to the first dose of the study treatment, excluding topical glucocorticoids for intranasal, inhalation or other routes of administration, or physiological doses of systemic glucocorticoids (i.e., no more than 10 mg/day prednisone or equivalent of other glucocorticoids);

4. Known or suspected active autoimmune diseases (congenital or acquired), such as interstitial pneumonia, uveitis, enteritis, hepatitis, hypophysitis, vasculitis, nephritis, thyroiditis, etc. (patients with vitiligo or complete remission of asthma in childhood, without requiring any intervention after adulthood can be enrolled; patients with type I diabetes well controlled by insulin can also be enrolled);

5. Known allotransplant (excluding corneal transplantation) or allogeneic hematopoietic stem cell transplantation;

6. Allergy to any ingredient of the monoclonal antibody;

7. Current interstitial lung disease;

8. Presence of other uncontrolled serious medical conditions, including but not limited to:

−Active or clinically uncontrolled severe infection;

−HIV-infection (HIV antibody positive);

−Acute or chronic active hepatitis B (HBsAg positive and HBV DNA > 1 x 10^3^/mL) or acute or chronic active hepatitis C (HCV antibody positive and HCV RNA > 15 IU/mL);

−Active pulmonary tuberculosis;

−Class III-IV congestive heart failure (New York Heart Association classification), poorly controlled and clinically significant arrhythmia;

−Uncontrolled arterial hypertension (systolic blood pressure ≥ 160 mmHg or diastolic blood pressure ≥ 100 mmHg);

−Any arterial thrombosis, embolism or ischemia within 6 months prior to inclusion for treatment, such as myocardial infarction, unstable angina, cerebrovascular accident or transient ischemic attack;

−Disease requiring anticoagulation with warfarin (coumarin);

−Uncontrolled hypercalcemia (calcium ion > 1.5 mmol/L or calcium greater than 12 mg/dL or corrected serum calcium > ULN), or symptomatic hypercalcemia requiring continued bisphosphonate therapy;

−Other malignant tumors (excluding cancer that has been cured, such as cervical carcinoma in situ, non-melanoma skin cancer, etc.);

9. Other acute or chronic diseases, psychiatric disorders or abnormal laboratory values, which may result in an increased risk associated with study participation or use of the study drug, or interfere with the interpretation of study results and render the patient ineligible for the study in the investigator’s judgment;

10. Pregnant or breastfeeding women.

Table S All pathological and metabolic findings after neoadjuvant sintilimab of enrolled patients

| **Table S All pathological and metabolic findings after neoadjuvant sintilimab of enrolled patients** | | | | | | | | | | | | | | | | | | | | | | | | | | | | | | | | | | | | |
| --- | --- | --- | --- | --- | --- | --- | --- | --- | --- | --- | --- | --- | --- | --- | --- | --- | --- | --- | --- | --- | --- | --- | --- | --- | --- | --- | --- | --- | --- | --- | --- | --- | --- | --- | --- | --- |
| **Patients**  **no.** | **1** | **2** | **4** | **5** | **9** | **10** | **11** | **12** | **13** | **14** | **15** | **16** | **17** | **18** | **19** | **20** | **21** | **22** | **23** | **24** | **25** | **26** | **27** | **28** | **29** | **30** | **31** | **32** | **33** | **34** | **35** | **36** | **37** | **38** | **39** | **40** |
| **Pathological regression (%)** | 30 | 20 | 5 | 60 | 40 | 10 | 95 | 90 | 40 | 100 | 40 | 95 | 20 | 100 | 60 | 20 | 10 | 50 | 95 | 95 | 100 | 0 | 60 | 30 | 40 | 35 | 100 | 95 | 100 | 90 | 20 | 10 | 10 | 5 | 95 | 45 |
| **Metabolic responds** | SMD | SMD | SMD | SMD | SMD | SMD | PMR | PMR | SMD | PMR | SMD | PMR | SMD | PMR | PMD | SMD | SMD | SMD | PMR | PMR | PMR | PMD | SMD | SMD | SMD | SMD | PMR | PMR | PMR | PMR | SMD | SMD | SMD | SMD | PMR | SMD |
| Note. - The patients of no.3 (100% pathological regression) and no.8 (95% pathological regression) were not underwent baseline PET-CT in our hospital, so we excluded them in this study. The patients of no.6 (SMD) and no.7 (SMD) were not underwent complete tumor resection, and we can't analysis the pathological regression. Therefore, the patients of no.6 and no.7 were excluded in this study. The patients of no.26 was classified as PMD according to PERCIST and had a new lesion on pleural highly suspected metastases. The pleural biopsy confirmed this patient had a real progression. Therefore, we did not exclude this case, and pathological regression was recorded as 0. | | | | | | | | | | | | | | | | | | | | | | | | | | | | | | | | | | | | |
